# Unlocking the heterogeneity of pediatric obesity: a phenotypic subtype-based paradigm for precision management

**DOI:** 10.3389/fendo.2025.1707952

**Published:** 2026-01-05

**Authors:** Yi Zhang, Rongi Zhou, Jiaqi Zhang, Xiaolu Yu, Jie Zhang

**Affiliations:** 1 Department of Pediatrics, First Affiliated Hospital, Henan University of Chinese Medicine, Zhengzhou, Henan, China; 2 School of Pediatrics, Henan University of Chinese Medicine, Zhengzhou, Henan, China

**Keywords:** childhood obesity, heterogeneity, phenotypic subtype, precision medicine, body mass index (BMI), metabolic syndrome, inflammation

## Abstract

Childhood obesity has emerged as a major global public health crisis. Current assessment methods, primarily relying on Body Mass Index (BMI), significantly limit the effectiveness of risk stratification and treatment due to their inability to capture the notable clinical heterogeneity of this condition. To address this, this review aims to propose a conceptual framework for pediatric obesity phenotypic subtypes, transcending BMI and rooted in dominant pathophysiological mechanisms, thereby offering a new theoretical basis for understanding its heterogeneity and advancing personalized medicine. Within this framework, we categorize childhood obesity into four core subtypes: 1) the “Dysmetabolic Subtype,” characterized by insulin resistance and ectopic fat deposition; 2) the “Inflammatory Subtype,” dominated by chronic, low-grade systemic inflammation; 3) the “Neurobehavioral Subtype,” originating from central appetite and reward system dysfunction; and 4) the “Biomechanical/Structural Subtype,” primarily driven by excessive mechanical load. This paper elaborates on the biological mechanisms, clinical identification pathways, key differential diagnostic points, and associations with specific long-term disease risks for each subtype. We believe that this phenotypic subtype framework provides a clear model for interpreting the diverse clinical outcomes and disease trajectories of childhood obesity. Adopting this multidimensional, multipath paradigm is a crucial step from the “one-size-fits-all” traditional management model towards a new era of precise risk assessment and personalized, “subtype-specific” treatment, which holds significant importance for improving long-term health outcomes.

## 1 Introduction

Childhood obesity has become one of the most severe global public health challenges of the 21st century. Statistics indicate a continuous rise in the prevalence of overweight and obesity among children worldwide, posing a significant public health concern (1, 2). Obesity not only threatens children’s current health but also portends a future fraught with health risks, including a significantly increased risk of developing type 2 diabetes, cardiovascular diseases, certain cancers, and psychosocial disorders (3).

Currently, clinical diagnosis and grading of childhood obesity primarily rely on the Body Mass Index (BMI). However, BMI, as a crude measure of body size, has increasingly revealed its inherent limitations (4). It cannot accurately differentiate between fat mass and muscle mass, nor can it reflect the distribution and metabolic activity of body fat. Crucially, BMI fails to adequately capture the high heterogeneity inherent in childhood obesity. At the same BMI level, individuals may exhibit vastly different metabolic health statuses, inflammatory levels, and long-term disease risks. This “same disease, different manifestations” phenomenon is a key reason why current “one-size-fits-all” intervention strategies (e.g., universal dietary and exercise recommendations) yield limited efficacy.

Given these limitations, the medical community urgently requires a new paradigm that transcends BMI to gain a deeper understanding of the complexity of childhood obesity. This paper proposes that childhood obesity should be viewed as a syndrome comprising various distinct phenotypic subtypes, each driven by its unique underlying pathophysiological mechanisms.

Therefore, the core objectives of this descriptive review are: 1) to systematically review and integrate interdisciplinary evidence; 2) to propose a conceptual framework for phenotypic subtyping of childhood obesity; and 3) to elucidate the intrinsic links between each subtype and long-term pathological development. The significance of this study lies in promoting a paradigm shift from “treating obesity” to “managing specific obesity subtypes,” thereby laying the theoretical foundation for precise risk stratification and personalized treatment of childhood obesity, and pointing directions for future research.

## 2 Limitations of current assessment methods: A “one-size-fits-all” approach in a heterogeneous disease

Before delving into the new subtyping framework, it is imperative to first clarify the inherent limitations of the current standard assessment method—Body Mass Index (BMI). When confronted with the highly heterogeneous pathological state of childhood obesity, BMI’s singular assessment characteristic proves inadequate for individualized risk assessment. Its primary limitations include: BMI’s inability to differentiate body composition, particularly its disregard for regional fat distribution (especially high-risk visceral fat), thereby rendering it insufficient in predicting an individual’s future metabolic risk. This leads to the clinical existence of numerous “metabolically healthy obese (MHO)” and “metabolically unhealthy normal weight (MONW)” individuals, posing significant challenges for early intervention and resource allocation (3). Therefore, there is an urgent need for a classification system that transcends anthropometric measurements and directly addresses the core pathophysiology.

## 3 Phenotypic subtype framework of pediatric obesity: classification based on dominant pathophysiological mechanisms

To comprehend the heterogeneity of childhood obesity, it is crucial to recognize that obesity is not merely a simple energy overload, but rather a differentiated manifestation of decompensation in various physiological systems under energy burden. The phenotypic subtype framework we propose is rooted in this understanding: it classifies a child’s obese state based on the system that first or predominantly develops dysfunction. Crucially, this functional approach is what distinguishes pathological fat accumulation from the normal increases in adiposity that occur during healthy growth and development. The classification into a subtype is not triggered by exceeding a certain threshold of body fat percentage, but rather by the emergence of objective evidence of systemic dysfunction. For instance, a child is categorized into the ‘Dysmetabolic Subtype’ not simply because they have a high BMI, but because they exhibit verifiable signs of metabolic decompensation, such as insulin resistance or dyslipidemia. This principle ensures that children undergoing normal developmental changes without associated pathology are not misclassified. In other words, the classification criterion is the “dominant pathophysiological pathway.” This framework attributes complex clinical manifestations to several relatively independent yet interconnected dominant pathological pathways, thereby providing clear targets for understanding and intervention. To clearly compare the characteristics of these subtypes, we summarize their core pathophysiology, clinical manifestations, and long-term risks in **Table 1**.

**Table 1:** Phenotypic subtype framework of pediatric obesity based on dominant pathophysiological mechanisms: a comprehensive comparison.

Phenotypic subtype	Core pathophysiological feature	Key biological mechanisms	Key clinical & laboratory manifestations	Primary long-term pathological risks	Key references
Dysmetabolic	Insulin resistance and ectopic fat deposition	Lipotoxicity; free fatty acid overflow; impaired insulin signaling in liver and muscle	Acanthosis nigricans; hyperinsulinemia; dyslipidemia (TG↑, HDL↓); elevated liver enzymes; HOMA-IR index↑	Type 2 diabetes; metabolic dysfunction-associated steatohepatitis (MASH)	(1, 3, 5, 6–11)
Inflammatory	Chronic, low-grade systemic inflammation	Adipokine dysregulation (e.g., TNF-α↑, IL-6↑); adipose tissue macrophage infiltration and M1 polarization	High-sensitivity C-reactive protein (hs-CRP)↑; interleukin-6 (IL-6)↑; other inflammatory factor elevation	Accelerated vascular aging; early-onset cardiovascular disease	(11–25)
Neurobehavioral	Central appetite dysregulation and hedonic eating	Hypothalamic leptin resistance; mesolimbic dopamine reward system “hijacked” by high-calorie foods	Binge eating/emotional eating; craving for specific foods; often accompanied by psychological issues (e.g., ADHD, anxiety)	Refractory obesity (as a manifestation of the underlying neurobehavioral disease); severe eating disorders; exacerbated comorbidities due to persistent energy imbalance	(26–32)
Biomechanical/Structural	Damage to body structure from excessive mechanical load	Excessive growth plate pressure; upper airway soft tissue compression leading to lumen narrowing	Orthopedic deformities (e.g., genu valgum, slipped capital femoral epiphysis); obstructive sleep apnea (OSA); snoring	Early-onset osteoarthritis; chronic pain; OSA-related cardiovascular and cerebrovascular complications	(33–49)

### 3.1 The dysmetabolic subtype

The core pathology of this subtype lies in the imbalance of energy metabolism homeostasis, primarily manifesting as insulin resistance (IR) and ectopic fat deposition. When energy surplus persists and the storage capacity of adipocytes is exceeded, “lipotoxicity” ensues (5). Excessive free fatty acids (FFAs) overflow and are abnormally deposited in key metabolic organs such as the liver, skeletal muscle, and pancreas. These ectopic fat depositions not only exacerbate hepatic steatosis but also directly interfere with insulin signaling pathways through mechanisms like activating inflammatory pathways, inducing oxidative stress, and mitochondrial dysfunction, leading to impaired glucose uptake and utilization by target organs (e.g., liver and muscle). Clinically, children with this subtype often present with typical features such as acanthosis nigricans, dyslipidemia (high triglycerides, low HDL-C), and elevated transaminases. They are at the highest risk of developing type 2 diabetes and severe liver disease in the future.

### 3.2 The inflammatory subtype

The pathological process of this subtype is driven by immune system dysregulation, predominantly characterized by chronic, low-grade systemic inflammation. Its core lies in the visceral adipose tissue gradually transforming into a pro-inflammatory center, where adipocytes and infiltrated M1 macrophages synergistically release large amounts of pro-inflammatory mediators (e.g., TNF-α, IL-6) into the bloodstream (13). This sustained inflammatory response poses a potential threat to the systemic vascular system, serving as a key driving factor for accelerated vascular aging and early-onset atherosclerosis. Therefore, even with normal early metabolic indicators, children with this subtype remain at a higher risk of future cardiovascular events.

### 3.3 The neurobehavioral subtype

The root cause of this subtype lies in central nervous system dysfunction, primarily manifested as central appetite dysregulation and hedonic eating behavior. On one hand, the hypothalamic homeostatic system may develop “leptin resistance,” leading to the brain’s inability to effectively receive satiety signals, thereby triggering persistent hunger and increased energy intake (28). On the other hand, high-sugar and high-fat foods excessively activate the mesolimbic dopamine reward system, inducing strong feelings of pleasure and craving, causing eating behavior to deviate from physiological needs and evolve into an addictive pattern driven by the pursuit of pleasure and stress relief (26). The primary long-term risk is the development of a refractory disease state, characterized by persistent failure to achieve energy balance due to the underlying neurobehavioral dysregulation. This state manifests clinically as severe, difficult-to-treat obesity (high BMI) and eating disorders, which in turn serve as the powerful engine driving the early onset and severity of all other obesity-related comorbidities (e.g., metabolic, cardiovascular).

### 3.4 The biomechanical/structural subtype

The pathological development of this subtype is primarily driven by damage to body structures caused by excessive mechanical load. Excessive mechanical load exerts immense pressure on children’s growth plates, which can lead to permanent orthopedic deformities such as genu varum or slipped capital femoral epiphysis (SCFE), severely impacting children’s motor function and quality of life (50). Concurrently, fat deposition in the upper airway can trigger obstructive sleep apnea (OSA). The intermittent hypoxia and stress response caused by OSA are not only independent risk factors for cardiovascular diseases but also exacerbate obesity itself by affecting energy metabolism and the secretion of appetite-regulating hormones (e.g., leptin and growth hormone), forming a vicious cycle (34).

## 4 Clinical identification and differential diagnosis of phenotypic subtypes

### 4.1 Constructing diagnostic pathways from clinical clues

Subtype identification begins with the keen recognition of clinical clues that transcend BMI. For instance, acanthosis nigricans on the neck and axillae is a strong indicator of the “Dysmetabolic Subtype”; while loud snoring and daytime sleepiness strongly suggest obstructive sleep apnea (OSA) within the “Biomechanical/Structural Subtype.” These “Clinical Cues” serve as triggers for initiating targeted diagnostic pathways. A structured diagnostic pathway should build upon these preliminary clues, guiding subsequent laboratory and auxiliary examinations to translate clinical impressions into objective diagnoses, thereby avoiding unnecessary or generalized testing.

### 4.2 Application and limitations of biomarkers in subtype stratification

Biomarkers provide objective evidence for subtype stratification, but a significant challenge is the well-recognized lack of standardized, pediatric-specific diagnostic cut-offs for many key indicators. Their maturity and utility vary significantly across subtypes. For the “Dysmetabolic Subtype,” clinical practice possesses mature diagnostic tools, such as HOMA-IR index and lipid profiles, making its diagnostic pathway the clearest. However, even here, there is no single, universally accepted definition for pediatric metabolic syndrome, with different organizations proposing varying component cut-offs (51). In contrast, identifying the “Inflammatory Subtype” presents greater challenges. Although high-sensitivity C-reactive protein (hs-CRP) is widely used, its non-specificity is high, and no consensus exists on a specific cut-off value that defines chronic, obesity-related inflammation in a child (52). Therefore, a panel of inflammatory biomarkers including hs-CRP, IL-6, adiponectin, and ferritin may hold higher diagnostic value, but this requires further standardization studies (13). The “Neurobehavioral Subtype” is currently the area most lacking reliable biomarkers. Its diagnosis primarily relies on validated psychological and behavioral assessment scales and detailed behavioral interviews. Although research is exploring the potential of postprandial hormone response curves or functional neuroimaging, these remain in the exploratory stage (28). Lastly, the diagnostic logic for the “Biomechanical/Structural Subtype” differs; it does not rely on circulating biomarkers but rather on functional or structural examinations with established categorical criteria. For example, obstructive sleep apnea is diagnosed based on an apnea-hypopnea index (AHI) from polysomnography, for which pediatric-specific diagnostic thresholds are well-established by clinical practice guidelines (53). The challenge here lies more in the clinical vigilance and timely referral awareness of clinicians.

A more fundamental challenge across subtypes, particularly for the inflammatory and dysmetabolic phenotypes, is the inherent limitation of a single biomarker measurement without knowledge of an individual’s pre-obesity baseline. Furthermore, standard diagnostic tests cannot capture all early pathological processes, such as initial immune cell infiltration into adipose tissue. In clinical practice, therefore, tracking the trend of biomarkers over time through longitudinal monitoring often provides more meaningful insight into disease progression than a single measurement. Our proposed framework is not static; it is designed to incorporate future, more sensitive and specific biomarkers as they transition from research to clinical practice, thereby continuously improving its precision.

### 4.3 Differential diagnosis and clinical reasoning for phenotypic overlap

Strictly categorizing every child into a single “box” is unrealistic. Phenotypic overlap is commonly observed in clinical practice, necessitating complex differential diagnostic reasoning. The core objective is not “forced classification,” but rather to identify the “dominant subtype” that poses the greatest and most urgent threat to the child’s health at the current stage. For example, a child with moderate insulin resistance and severe OSA, despite exhibiting “Dysmetabolic Subtype” features, faces a more immediate threat from the nocturnal hypoxia and sudden death risk associated with severe OSA, making the “Biomechanical/Structural Subtype” the priority for intervention. Therefore, the culmination of clinical reasoning is to formulate a prioritized intervention plan, addressing the most critical or urgent issues first, followed by other relatively secondary concerns.

## 5 Discussion

The central argument of this review is that a phenotypic subtype framework, based on dominant pathophysiological mechanisms, can provide a deeper understanding and facilitate a more effective response to the heterogeneity of childhood obesity. In alignment with the challenges outlined in the introduction, this section aims to delve into the inherent logic, clinical interpretation, and significant implications of this framework for reshaping disease perception, rather than reiterating future research blueprints.

### 5.1 Distinguishing pathological dysfunction from normal developmental adiposity

A critical challenge in pediatric obesity is distinguishing pathological adiposity from the physiological increases in fat mass inherent to normal growth and puberty. Conventional BMI-based assessments often fail at this juncture, creating clinical ambiguity. The subtype model proposed herein is designed to resolve this issue by shifting the diagnostic focus from simple anthropometry to the underlying pathophysiology.

The core principle of this framework is that classification requires objective evidence of “systemic decompensation.” A child with a high BMI who maintains metabolic homeostasis—presenting with normal insulin sensitivity, lipid profiles, and inflammatory markers—would not be classified into the Dysmetabolic or Inflammatory subtypes, despite their level of adiposity. In this model, classification is only applied when the body’s capacity to safely store excess energy is overwhelmed, leading to measurable downstream consequences.

For instance, the trigger for the Dysmetabolic Subtype is not a specific BMI value, but rather the clinical manifestation of lipotoxicity, such as the appearance of acanthosis nigricans or laboratory findings of elevated HOMA-IR. Similarly, the Biomechanical/Structural Subtype is identified by evidence of structural damage, such as genu valgum or diagnosed obstructive sleep apnea. These are clear pathological signals, distinct from the changes associated with normal growth.

In essence, this framework provides clinicians with a structured method to determine if a child’s adiposity is causing functional harm. This approach avoids the over-medicalization of children with constitutionally larger body sizes while precisely identifying those who have crossed the threshold from a state of energy surplus to one of active, subtype-specific disease.

### 5.2 Practical implementation and a tiered diagnostic approach

The practical implementation of this subtype framework in real-world clinical settings, particularly in primary care, presents valid challenges regarding cost, time, and the availability of specialized technology. We acknowledge that “gold-standard” assessments for each subtype, such as MRI for ectopic fat quantification or functional neuroimaging, are not feasible for routine screening. To address this, we advocate for a tiered diagnostic approach that balances precision with practicality, a strategy consistent with current clinical practice guidelines for managing comorbidities in pediatric obesity (54, 55). The Endocrine Society, for instance, explicitly recommends that screening for comorbidities be applied in a ‘hierarchal, logical manner’ (54). This stepwise model ensures that resources are allocated efficiently:

Tier 1: Initial Screening in Primary Care. The foundation of this framework relies on low-cost, high-yield tools accessible to all clinicians. Practical proxy measures are invaluable at this stage. For example, while MRI is the gold standard for ectopic fat, simple anthropometric indices like the Waist-to-Height Ratio (WHtR) can serve as effective and practical proxies for central adiposity and are strong predictors of cardiometabolic risk, often superior to BMI (56). These indices, combined with a detailed clinical history and standard laboratory panels (e.g., fasting lipids, glucose, ALT), can provide strong preliminary evidence to suspect a dominant subtype.

Tier 2: Targeted Specialist Referral and Assessment. A referral for more specialized testing is initiated only when Tier 1 findings raise a “red flag.” At this stage, composite risk scores, such as a continuous metabolic syndrome score calculated from standard lab values, can offer a more integrated and dynamic assessment of metabolic risk than single markers alone. A child with significant snoring and reported apnea would be referred for polysomnography, which is the standard of care. Similarly, a baseline high-sensitivity C-reactive protein (hs-CRP) can be used to screen for an inflammatory phenotype before considering more extensive cytokine panels.

Tier 3: Advanced Research and Complex Cases. The most technically demanding and expensive investigations, such as detailed metabolomics, advanced neuroimaging, or comprehensive inflammatory marker arrays, should be reserved for tertiary care centers, complex diagnostic dilemmas, and research settings. This includes advanced platforms like NMR-based metabolomics, which can identify novel metabolite signatures associated with specific obesity subtypes and future cardiometabolic risk, paving the way for the discovery of new biomarkers. This tiered strategy inherently mitigates the challenge of unknown individual baselines and undetectable early pathology, as it prioritizes the management of objectively measured, extant dysfunction.

By adopting this tiered strategy, the framework provides a practical clinical roadmap rather than an unmanageable list of required tests. It guides clinicians to use existing resources to make a probable subtype diagnosis and to rationalize the use of advanced diagnostics, making precision-based care more achievable in diverse healthcare environments.

### 5.3 Interplay of phenotypic subtypes and clinical complexity: from overlap to prioritization

The four subtypes proposed in this paper serve as theoretical models established to elucidate the primary pathological pathways. In clinical reality, they are not entirely separate “islands” but exhibit significant interactions and overlaps, which is crucial to understand and does not invalidate the framework. On the contrary, recognizing this complexity is a core strength of a pathophysiology-based model. The clinical utility of this framework lies not in achieving a pristine, mutually exclusive categorization, but in identifying the dominant or most pressing pathological pathway at a given time. This allows clinicians to triage interventions and allocate resources effectively. For instance, the “Inflammatory Subtype” is a significant accelerator of the “Dysmetabolic Subtype,” as pro-inflammatory factors can directly worsen insulin resistance(Liang, 5). However, a child presenting with moderate insulin resistance but severe obstructive sleep apnea (OSA, a Biomechanical/Structural driver) requires immediate prioritization of the OSA due to its acute risks of cardiovascular stress and neurocognitive impairment. The “Neurobehavioral Subtype” often acts as the “energy engine” driving all other subtypes; if uncontrolled, it can undermine interventions targeting other domains. Thus, the framework provides a structured mental model for navigating this complexity, transforming an overwhelming list of comorbidities into a manageable hierarchy of treatment priorities based on the dominant pathophysiology.

### 5.4 Clinical decision prioritization and window-of-opportunity intervention based on subtype characteristics

The most significant clinical value of this framework lies in guiding clinicians, under existing medical conditions, to formulate more targeted and urgent monitoring and intervention priorities. This is not merely a simple task assignment but is based on a profound understanding of the unique pathological trajectories and potential harms of different subtypes, thereby seizing the “treatment window” before irreversible damage occurs.

When clinical evidence points to the “Dysmetabolic Subtype,” such as the discovery of acanthosis nigricans, its clinical significance extends far beyond aesthetic concerns. Acanthosis nigricans is a cutaneous marker of hyperinsulinemia, and its appearance signifies that insulin resistance has transitioned from an abstract “risk” to an active, ongoing pathophysiological process that continuously exerts pressure on β-cell function. Therefore, immediately initiating baseline assessment and regular monitoring of fasting blood glucose, insulin, and lipid profiles becomes the highest priority clinical action. This is not about preventing future problems but managing a countdown to type 2 diabetes that has already begun.

Similarly, when an obese child complains of joint pain or is observed to have severe snoring, this framework guides us to consider these as potential core manifestations of the “Biomechanical/Structural Subtype” and to immediately initiate specialist referral. The clinical reasoning behind this is based on a prudent weighing of “risk-benefit.”

Regarding joint pain: In obese children, this should never be easily attributed to “growing pains” or “general discomfort due to excess weight.” It is a strong signal that requires urgent exclusion of slipped capital femoral epiphysis (SCFE) or Blount’s Disease (tibia vara). Particularly for SCFE, its mechanism involves excessive mechanical shear forces acting on the vulnerable growth plate of the femoral head during adolescence, leading to femoral head displacement. This is not simply arthritis but an orthopedic emergency that can lead to avascular necrosis of the femoral head and lifelong disability (50). The responsibility of frontline clinicians is not to diagnose but to maintain high vigilance and immediately refer the child to a pediatric orthopedic surgeon. Orthopedic surgeons can rapidly make a diagnosis through professional physical examination and X-ray imaging, and once confirmed, urgent surgical fixation may be required to salvage hip joint function. Any delay in observation could lead to missing the optimal treatment window, resulting in irreversible damage.

Regarding severe snoring: This is by no means a harmless noise issue but a typical clinical manifestation of obstructive sleep apnea (OSA). Its pathological core is the recurrent collapse of the upper airway during sleep, leading to severe physiological events of intermittent hypoxia and sleep architecture disruption. This nocturnal hypoxia places a tremendous burden on the developing cardiovascular system, serving as an independent risk factor for childhood hypertension and cardiac remodeling; concurrently, fragmented sleep can severely impair neurocognitive functions related to learning, memory, and attention (57, 58). Therefore, immediate referral of the child to a sleep medicine center for polysomnography (PSG) is the gold standard for assessing its severity and initiating treatment (e.g., CPAP). Ignoring this sign is tantamount to tacitly allowing the child’s circulatory and nervous systems to be exposed to the risks of hypoxia and stress throughout the night.

Thus, the “referral” guided by this framework is not a simple procedural operation but a critical clinical decision based on a profound understanding of the potential severity of pathology and irreversible consequences, aimed at seizing the treatment window. This decision-making model shifts the clinical focus from “how severe is the problem?” to “what is the nature of the problem?”, thereby truly achieving precision and foresight in intervention.

### 5.5 Reshaping disease trajectory perception: from “single-line” to “multi-path” model

Traditional perceptions tend to depict the consequences of obesity as a linear, progressive process. This framework, through the discussions in the text, proposes a “Multi-path” disease trajectory model. This model posits that children of different subtypes, starting from the “obesity” origin, travel along several distinct highways towards their respective pathological endpoints: the “Metabolic type” leads to early-onset type 2 diabetes; the “Inflammatory type” leads to cardiovascular events in youth; and the “Biomechanical type” leads to loss of physical function and decreased quality of life. This multi-path perception can explain the diverse outcomes observed clinically and emphasizes the importance of early subtyping for predicting which “pathway” carries the highest risk. It shifts our focus from “how obese is this child?” to “which dangerous pathway is this child’s obesity progressing along?”, thereby deepening our understanding of long-term risks. Future research should focus on several aspects: firstly, the discovery and validation of subtype-specific biomarkers are needed, particularly for the neurobehavioral and inflammatory driven subtypes, to achieve more precise early diagnosis and risk stratification. Secondly, the development and clinical validation of personalized intervention strategies based on these subtypes are crucial, including pharmacological, lifestyle, and behavioral therapies. Furthermore, integrating multi-omics technologies such as genomics, proteomics, and metabolomics will aid in a more comprehensive understanding of the complex pathophysiological networks of each subtype. Finally, large-scale, long-term cohort studies are essential to further clarify the causal associations and disease trajectories between each subtype and different long-term complications, providing solid evidence for achieving precision medicine in childhood obesity.

### 5.6 From subtype to strategy: guiding clinical application

The primary clinical value of this framework lies in its ability to guide a structured, proactive management plan that extends beyond generic advice. It encourages a shift from a passive, BMI-centric screening model to an active, clue-based approach where clinicians are prompted to screen for subtype-specific signals like acanthosis nigricans (Dysmetabolic) or snoring (Biomechanical/Structural). The identification of these clues is, in itself, a more powerful form of risk stratification than BMI alone because it defines the *nature* of the patient’s primary risk. For example, a child with a dominant Dysmetabolic subtype is immediately stratified as high-risk for type 2 diabetes and NAFLD, while another with an Inflammatory subtype is flagged for accelerated atherosclerotic risk, regardless of their current lipid levels.

This precise, mechanism-based risk stratification is the foundation for truly personalized intervention. Management strategies can be tailored to target the dominant underlying pathophysiology. A child with a dominant Dysmetabolic subtype would benefit most from interventions focused on improving insulin sensitivity, such as a low-glycemic-index diet, which contrasts sharply with the needs of a Neurobehavioral subtype, for whom behavioral therapies like Cognitive Behavioral Therapy (CBT) are paramount. Likewise, a child identified with a Biomechanical/Structural subtype requires immediate referral to specialists like orthopedics or sleep medicine, and their exercise plan would be customized to include non-weight-bearing activities like swimming to protect vulnerable joints—a critical nuance missed by generic advice. By translating a broad diagnosis of “obesity” into a specific, mechanistically-defined subtype, this framework provides a practical and actionable paradigm for delivering truly personalized and preventative care in real-world pediatric settings.

To clarify the distinction between current practice and our proposed framework, the following table (**Table 2**) provides a direct comparison of the clinical management approach for two illustrative scenarios:

**Table 2:** Contrasting current practice with a phenotypic subtype-directed management strategy.

Clinical scenario	Current standard practice (Problem-list based)	Subtype-directed strategy (Mechanism-based)	Core difference
Obesity + Type 2 Diabetes	Manage obesity (generic diet/exercise) and manage T2D (e.g., with metformin). Two problems are addressed in parallel.	Identify as “Dysmetabolic Subtype” dominant. Intervention is focused on intensively improving insulin sensitivity: a low-glycemic load diet is paramount, and pharmacotherapy prioritizes agents like GLP-1 RAs that target both weight and glycemia.	Nature of Intervention: Diet is mechanistically tailored, not generic. Drug choice is aligned with the dominant pathophysiology (insulin resistance/ectopic fat).
Obesity + Joint Pain	Manage obesity (generic advice) and refer to orthopedics. Two problems are addressed in parallel.	Identify as “Biomechanical Subtype” dominant. Expedited orthopedic referral is urgent to rule out SCFE. Crucially, the physical activity prescription is immediately and fundamentally altered to exclusively non-weight-bearing exercise (e.g., swimming, cycling) to protect joints while promoting energy expenditure.	Sequence & Specificity: Recognizes the urgency of the structural issue. Exercise is not just “prescribed” but is specifically engineered to avoid harm, a nuance often missed in generic advice.
Obesity + Binge Eating	Often overlooked, or the patient receives generic dietary counseling which is often ineffective.	Identify as “Neurobehavioral Subtype” dominant. The first-line intervention shifts from a specific diet plan to referral for cognitive behavioral therapy and assessment for anxiety/depression. Pharmacotherapy considers agents targeting appetite and reward.	Root Cause Focus: Recognizes the driver is in the brain, not the plate. Addresses the behavioral and psychological pathology first to enable subsequent metabolic interventions.

As illustrated, the key difference lies in moving from a problem-list approach, where comorbidities are managed in parallel, to a mechanism-based approach, where the dominant pathophysiology dictates the priority, nature, and specificity of the entire treatment plan. This shifts the clinical question from “What problems does this child have?” to “What is the driving mechanism behind these problems, and how do we target it first and most effectively?”

### 5.7 The influence of puberty as a confounding and modifying factor

The assessment of childhood obesity and its subtypes must be interpreted within the dynamic context of puberty. This period of profound hormonal change significantly influences body composition and metabolic health, acting as both a potential confounder and a modifier of the proposed phenotypes. There is a well-documented state of physiological, transient insulin resistance that normally occurs during mid-puberty, driven primarily by an increase in growth hormone secretion. This presents a diagnostic challenge, as it must be carefully distinguished from the pathological insulin resistance that characterizes the Dysmetabolic subtype. Therefore, the interpretation of markers like HOMA-IR is not absolute and requires careful consideration of the child’s pubertal (Tanner) stage.

Furthermore, puberty can act as a critical window that modifies or accelerates a pre-existing risk. The sex-specific hormonal surges drive distinct changes in fat mass and distribution, which could exacerbate an underlying Dysmetabolic or Inflammatory predisposition. The significant neurobehavioral and emotional shifts inherent to adolescence can also interact with and potentially worsen a Neurobehavioral subtype. Consequently, the accurate application of this framework requires clinicians to integrate a child’s developmental stage into their assessment, viewing puberty as a period that can both mimic and magnify the underlying pathophysiological subtypes of obesity.

### 5.8 Addressing the fundamental role of energy surplus

It is crucial to emphasize that chronic positive energy balance is the necessary precondition for the development of all pediatric obesity subtypes presented in this framework. Our phenotypic stratification does not supersede the principle of managing energy intake and expenditure; rather, it is designed to refine it. The identification of a dominant subtype provides a mechanistic understanding to guide more effective and sustainable interventions targeting the root cause. For example, managing energy intake in a child with a dominant “Neurobehavioral Subtype” necessitates targeting central appetite dysregulation, whereas in a “Biomechanical Subtype,” the focus must include adapting physical activity to protect vulnerable structures. Thus, this framework enables a more precise approach to rectifying the fundamental energy imbalance.

### 5.9 Estimated subtype distribution and clinical relevance

A comprehensive understanding of any phenotypic framework requires consideration of the distribution of patients across the proposed categories. Data-driven phenotyping studies, which apply clustering algorithms to large pediatric cohorts, provide empirically derived estimates for subtype prevalence. For instance, a comprehensive review on the genetics of pediatric obesity indicates that monogenic forms, often manifesting as severe early-onset obesity with neurobehavioral features, account for approximately 7% of severe childhood obesity cases, with MC4R pathway mutations alone representing 3-5% of such cases (26). This aligns with the proposed ‘Neurobehavioral Subtype’ driven by central appetite dysregulation. Furthermore, clinical cohort studies highlight that children with severe obesity represent a distinct high-acuity subgroup, reporting significantly greater impairments in physical well-being and health-related quality of life compared to their overweight or obese peers (59). This underscores that the clinical relevance of a subtype is not exclusively determined by its prevalence. Subtypes with lower population frequency may warrant particular clinical attention due to the severity of their associated health outcomes. For instance, although slipped capital femoral epiphysis (SCFE) is relatively uncommon in the general pediatric obesity population, its identification through the “Biomechanical/Structural Subtype” framework is critically important, as a missed diagnosis can lead to irreversible joint damage and permanent disability. This concept is reinforced by a recent systematic review and meta-analysis, which suggests that all childhood obesity phenotypes, including those initially perceived as metabolically healthy, carry an elevated risk for cardiometabolic diseases in adulthood (60). Therefore, this classification system aims not only to address highly prevalent conditions but also to systematically identify and prioritize the management of high-acuity pathologies, regardless of their frequency, across the obesity spectrum.

### 5.10 The “at-risk” phenotype: managing severe adiposity without current subtype classification

The framework necessarily identifies children based on extant pathophysiological derangements. However, we fully acknowledge a critical point that a child with severe adiposity not yet meeting these criteria represents a high-risk state, not a healthy one. This situation, which often aligns with the concept of “metabolically healthy obesity,” should be conceptualized as “At-Risk Obesity.” The clinical imperative for these patients is not inaction, but rather the most vigorous pursuit of foundational lifestyle intervention to reverse the energy imbalance. Furthermore, this stage mandates proactive monitoring for the emergence of subtype-specific markers (e.g., annual screening for dyslipidemia, insulin resistance, and liver enzymes), as the appearance of conditions like MASLD is indeed a sign that intervention was needed earlier. Thus, the framework sharpens the focus on preventing the transition from “at-risk” to a defined, pathological subtype.

### 5.11 On the prevalence of “deceptively” high BMI and normal growth

A high BMI (e.g., >95th percentile) without elevated adiposity is uncommon, True “deceptively” high BMI, driven primarily by high lean mass in a muscular child, is indeed rare in the general pediatric population. The more common and clinically relevant scenario is the misclassification of metabolic risk by BMI alone. For instance, a large national study of US adolescents found that even among those with normal BMI, 9.8% had hepatic steatosis and 4.8% had prediabetes, confirming that BMI is an imperfect proxy for metabolic health (61). Conversely, not all children with a high BMI have adiposity-related complications; this group is often described as having “metabolically healthy obesity.” Our framework addresses both ends of this spectrum. The subtyping paradigm does not require a high BMI to be “deceptive” to be useful; rather, it is precisely because BMI is a poor indicator of pathophysiology that a mechanistic subtyping system is needed. The framework’s primary goal is to identify the dominant dysfunctional pathway, moving beyond the debate of BMI’s body composition correlates and directly assessing the clinical consequences of excess adiposity, when present.

## 6 Conclusion

Childhood obesity is a complex clinical syndrome characterized by high heterogeneity. A single BMI metric is far from sufficient to guide precise risk assessment and management. This review systematically proposes and discusses a phenotypic subtype framework based on dominant pathophysiological mechanisms, categorizing childhood obesity into dysmetabolic, inflammatory, neurobehavioral, and biomechanical/structural subtypes, and thoroughly explores the clinical challenges and diagnostic pathways for identifying these subtypes. This framework not only provides a clear theoretical model for understanding the biological basis of obesity heterogeneity but, more importantly, offers a clear and actionable path for clinical practice to transition from the traditional “one-size-fits-all” model to a new era of “subtype-specific” precision medicine. Adopting this multidimensional, multi-path cognitive and management model is crucial for enhancing intervention effectiveness, improving long-term health outcomes, and ultimately curbing this global epidemic.
